# Editorial for Special Issue: “Molecular Mechanisms Underlying Fatty Liver Disease: From Pathogenesis to Treatment, 2nd Edition”

**DOI:** 10.3390/cimb48030288

**Published:** 2026-03-09

**Authors:** Wei Guo, Haibo Dong

**Affiliations:** Center for Translational Biomedical Research, University of North Carolina at Greensboro, North Carolina Research Campus, Kannapolis, NC 28081, USA; w_guo2@uncg.edu

The nomenclature for fatty liver diseases, characterized by the accumulation of fat in the liver, has recently been updated by a global consensus of liver organizations and societies for steatotic liver disease (SLD). This disease includes metabolic dysfunction-associated steatotic liver disease (MASLD; previously known as non-alcoholic fatty liver disease, NALFD), alcohol-associated liver disease (ALD), as well as a newer, blended category termed metabolic and alcohol-associated liver disease (MetALD), which accounts for individuals who have both metabolic risk factors and moderate alcohol consumption [1,2].

These clinical conditions share some common features, such as lipid accumulation, oxidative stress, and inflammatory response, but they are ultimately distinct, driven by different primary risk factors. The pathogenesis of MASLD is predominantly fueled by metabolic disturbances, notably lipid dyshomeostasis, insulin resistance, and systemic inflammation, while ALD, on the other hand, results from alcohol consumption, where alcohol metabolism induces inflammation, oxidative stress, and liver injury [3,4]. The prevalence of MASLD/ALD has increased over the past three decades, imposing significant economic burdens on health care systems, especially following the COVID-19 pandemic [5,6]. Therefore, continuous and focused investigations are essential to advance our understanding of the pathology and the underlying mechanisms targeting these fatty liver diseases.

This Special Issue of *Current Issues in Molecular Biology* (2nd Edition) includes seven research studies that cover diverse and unique aspects of MASLD/ALD, with emphasis on cellular and molecular mechanisms, alteration of lipid hormone signaling, examination of disease patterns in specific populations, as well as identification of innovative therapeutic interventions.

RNA methylation is essential for regulating RNA function and is involved in many diseases, including MASLD. However, the role of 5-methylcytosine (m5C) in the development of MASLD is unclear. Yang et al. [7] implemented RNA-Seq and RNA-BS-Seq analyses in a murine model of MASLD and showed that hypermethylated genes were mainly enriched in lipid metabolism pathways. Out of the 156 examined genes, 72 were upregulated and increased with m5C. This work indicates that m5C methylation may contribute to the development of MASLD, providing new avenues for potential therapeutic interventions.

Genetic factors play an important role in MASLD, with *PNPLA3* (adiponutrin/patatin-like phospholipase domain-containing protein 3) identified as a major susceptibility gene and *FTO* (fat mass and obesity-associated) polymorphisms strongly linked to obesity risk. While the prevalence of *PNPLA3* rs738409 and rs2896019 and *FTO* rs9939609 and rs17817449 has been reported globally, data from Mongolia are lacking. Tsedendorj et al. [8] found that the PNPLA3 rs738409 GC/GG genotype and FTO rs9939609 AT/AA genotype are strongly associated with an increased risk of MASLD, as individuals with a higher SNP number are more likely to develop MASLD. Therefore, genetic screening for these variants could help early identification of high-risk individuals, enabling targeted therapies to combat MASLD.

Endocrine disorders, including hypercortisolism, hypothyroidism and polycystic ovary syndrome (PCOS), promote MASLD through overlapping mechanisms. Dobre et al. [9] examines the complex role of disrupted lipid hormone pathways in the development of metabolic disorders, particularly MASLD. Their review contributes to our understanding of the overlapping pathophysiology between endocrine, hepatic, and reproductive disorders, highlighting the need for multidisciplinary approaches to optimize diagnosis, treatment, and long-term cardiometabolic outcomes.

In search of effective pharmacological therapies, Fu et al. [10] investigated the role of empagliflozin, a sodium-glucose cotransporter-2 inhibitor, in an animal model of MASLD. These researchers demonstrated that empagliflozin significantly reduced hepatic steatosis and improved liver injury by suppressing NF-κB-mediated inflammatory signaling and inhibiting fibrotic markers such α-SMA and COL1A1, while modulating TIMP-1 and MMP-9 expression. The findings highlight empagliflozin’s potential as a mechanism-based therapeutic strategy for treating MASLD.

Interestingly, Zeng et al. [11] utilized ultra-high-performance liquid chromatography–mass spectrometry (UHPLC-MS) combined with network pharmacology to identify the active ingredients and mechanisms of action of *Rhodiola crenulate* (RC), a traditional Chinese medicinal plant, in improving MASLD. They found that Catechin gallate, a major bioactive compound, exhibited a significant effect in reducing lipid accumulation in HepG2 cells. They further revealed that the therapeutic effects of Catechin gallate on fatty liver disease may involve a stable binding interaction towards target molecules such as ABCB1, DYRK1A, PGD, and FUT4. This study provides a theoretical foundation for the application of RC in treating MASLD.

MASLD has been reported to be associated with cardiometabolic diseases and mental health issues. Jurek et al. [12] evaluated microbiota-derived metabolites, such as bile acids and short-chain fatty acids (SCFAs), as potential biomarkers of depressive disorder (DD) in morbidly obese women undergoing bariatric surgery. They found that DD was associated with higher levels of specific metabolites, including glycodeoxycholic acid (GDCA) and propionate, which correlated with metabolic markers. But MASLD and metabolic syndrome rates did not differ between groups. Their findings suggest that GDCA and propionate may influence DD risk, but further investigations are needed to confirm the therapeutic potential of these biomarkers.

ALD remains a major cause of cirrhosis and liver-related mortality worldwide, yet effective therapies are limited, in part because immune dysregulation and metabolic injury are tightly intertwined. Dong et al. [13] provided an integrated, cell-type-resolved framework for how ethanol-driven DAMP and PAMP cues engage a coordinated network of interferon regulatory factors (IRFs) across hepatocytes and immune cells. In particular, the authors summarized how danger-signaling pathways—including TLR4, TLR9, and cGAS–STING—converge on key IRF nodes (e.g., IRF1, IRF3, IRF4, IRF5, IRF7, and IRF9) to shape inflammatory, metabolic, and cell-fate programs, and highlighted emerging therapeutic opportunities that converge on these circuits, such as STING/TBK1 inhibition, NETosis blockade, IL-22-based epithelial repair, and JAK–STAT modulation.

Collectively, these research works provide a comprehensive and insightful narrative of the interconnected mechanisms underlying the pathogenesis of MASLD/ALD and related disorders. This Special Issue underscores the necessity of multidisciplinary approaches to tackle the global burden of fatty liver disease.

We would like to extend our appreciation to all contributing authors for their valuable insights and to our reviewers for their rigorous and thoughtful evaluations, which have strengthened each manuscript. We hope this Special Issue not only advances our current understanding but also stimulates continued inquiry and fosters the development of transformative solutions for improving liver health worldwide.
